# Statistical Analysis of the Influence of Various Types of Graphite Precursors and Oxidation Methods on the Gas Sensor Properties of Reduced Graphene Oxide

**DOI:** 10.3390/s24196346

**Published:** 2024-09-30

**Authors:** Łukasz Drewniak, Sabina Drewniak, Marcin Sajdak, Roksana Muzyka

**Affiliations:** 1 Department of Optoelectronics, Faculty of Electrical Engineering, Silesian University of Technology, 44-100 Gliwice, Poland; lukasz.drewniak@polsl.pl; 2 Department of Air Protection, Faculty of Energy and Environmental Engineering, Silesian University of Technology, 44-100 Gliwice, Poland; marcin.sajdak@polsl.pl

**Keywords:** reduced graphene oxide (rGO), reduction, exfoliation, sonification, gas sensor, ANOVA

## Abstract

The fabrication process of reduced graphene oxide depends on many factors (e.g., graphite precursor, methods of oxidation, reduction, and exfoliation) which have a significant influence on the properties of this material. Therefore, their selection is not easy due to the large number of possible combinations of these factors. To overcome this problem, we proposed to use a multivariate analysis of variance method of finding associations between the qualitative type of independent variables and the quantitative type of dependent variable. Using ANOVA, we showed that the combination (interaction) of these variables is more important than the individual influence of the variables on the fabricated rGO. Knowing how the particular variables and their combinations affect the properties of rGO, it is easier to plan the fabrication process of this material. In this paper, we analyzed the number of oxide layers and designated the most promising oxides in terms of sensor gas application. Independently, we fabricated chemiresistor sensors and studied their response to NO_2_ in the analyzed atmosphere. We were able to combine the experimental results with statistical analysis indicating which oxidation methods and which graphite precursors will provide the best sensitivity.

## 1. Introduction

**Figure 1:**
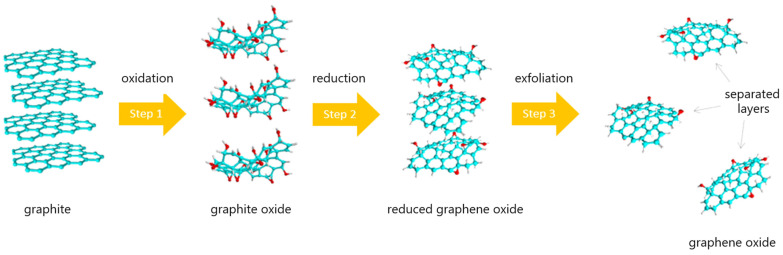
The preparations and chemical structures scheme of rGO.

Reduced graphene oxide (rGO) is a promising candidate for the developing of NO_2_ sensors due to its unique properties, such as good electrical conductivity at room temperature, high surface to volume ratio, high sensitivity, high flexibility, and numerous structural defects, including oxygen functional groups which can act as additional active sites for gaseous molecules [1,2,3,4]. rGO is a cheap material that can be relatively easily and quickly produced on a large scale. Generally, rGO is produced from graphite in three main steps: oxidation, reduction, and exfoliation (Figure 1). The oxidation can be performed in several ways. Some of the most popular ways include the Hummers, Staudenmeier, and Brodie methods [5,6,7,8]. The reduction can also be performed in several ways, for example, using flash photoreduction, thermal annealing, or hydrothermal, catalytic reduction [9,10,11,12]. For obtaining exfoliation, ultrasounds could be used [13]. Each of these steps are important because they affect the physicochemical properties of the rGO, and thus its further applications.

The oxidation process introduces oxygen functional groups between the graphite layers (epoxy (C-O-C), hydroxyl (OH), carboxyl (R-COOH), and carbonyl (C = O)), which basically increase the distance between the graphite layers and the hydrophilicity of the layers [6,14]. The hydroxyl (-OH) and epoxy (C-O-C) groups are located in the basal plane, while carboxylic groups (-COOH) are located mainly at the edges of the planes [15,16]. The graphene-like honeycomb lattice is preserved; however, the carbon atoms are disordered [6]. There are many methods of graphene oxidation. The most popular are Hummers’, Staudenmaier’s, Brodie’s, and Tours’ methods [6,17,18,19,20] and their various modifications.

Reduction removes functional groups and defects in an atomic-scale lattice and recovers the conjugated network of graphite lattice [6,21]. The degree of reduction depends on the method used. Generally, the reduction methods can be classified into three categories: chemical, thermal, and electrochemical [6]. The most popular are chemical methods because they can be easily applied on a large scale. Exfoliation reduces the number of layers to a few layers (few-layered stacks of rGO) or a monolayer [22]. This process can be performed in the liquid phase or by thermal or mechanical methods [23]. A detailed description can be found in [6]. Although the fabrication process of rGO with specific properties seems easy, it is not. This is because the process consists of many variables that have a significant influence on rGO properties [24]. This means that rGO can be produced in many ways and with different properties. For example, in paper [25], it has been shown that depending on the reduction method (chemical, hydrothermal, electrochemical, or thermal), materials with completely different characteristics can be obtained, such as sheet size, amount of remaining oxygen, defects, and electroactivity.

Therefore, in order to prepare rGO with specific properties, it is necessary to study the influence of the graphite precursor, and the oxidation, reduction, and exfoliation methods, as well as their combination (interaction), on the properties of this material. Unfortunately, this involves the production of a large number of structures that differ in terms of the graphite precursor, and the oxidation, reduction, and exfoliation methods used. Obviously, such an approach is ineffective and time consuming. To overcome this difficulty, we suggest using a multivariate analysis of variance (ANOVA) method, which allows us to determine the influence of the mentioned variables on one or more dependent variables (e.g., number of graphene layers in rGO, active sites, electrical conductivity, working temperature, etc.). Knowing how the independent variables are related to the dependent variables, it is easier to decide which precursor and which methods of oxidation, reduction, and exfoliation to choose to obtain a material with the desired properties.

In this paper, we analyze the influence of different types of precursors, oxidation methods, and their combination/interaction on the number of graphene layers in rGOs. We have shown which of the examined graphites and oxidation methods have the greatest impact on the number of graphene layers in rGO and whether the combination (interaction) of these variables is important. We also showed which configuration of the variables is the most suitable for preparation of rGO for sensor applications (e.g., for NO_2_ detection).

## 2. Materials and Methods

**Figure 2:**
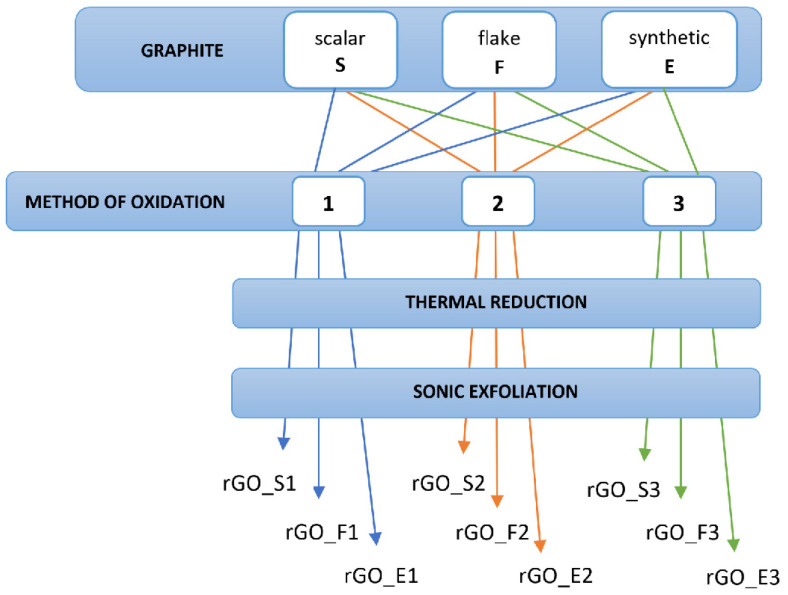
Scheme for the production of rGO samples analyzed in this paper. 1—modified Hummers’ method, 2—modified Hummers’ method, 3—modified Tour’s method.

We have prepared 9 samples of rGO produced using various graphite precursors and oxidation methods. We used three types of graphite precursors: flake with 82 nm grains, scalar (59 nm), and synthetic (50 nm), and three oxidation methods: two different varieties of Hummer’s method and one modified Tour’s method. The sample preparation scheme is shown in Figure 2.

In **step 1**, 1 g of graphite powder was added to oxidizing agents. The oxidants for method 1 were 20 mL of H_2_SO_4_, 15 mL of HNO_3_, and 3 g of KMnO_4_; for method 2, they were 30 mL of H_2_SO_4_, 3 g of NaNO_3_, and 3 g of KMnO_4_; and for method 3, they were 45 mL of H_2_SO_4_, 5 mL of H_3_PO_4_, 1.5 g of KNO_3_, and 5 g of KMnO_4_. Then, 100 mL of deionized water and 60 mL of 3% H_2_O_2_ were added and stirred for over 30 min. After that, the mixture was centrifuged at 5000 rpm (0.5 h/cycle), decanted, washed in a 10% HCl solution, centrifuged, and washed again in water with a conductivity of 0.14–0.18 µS until it obtained a pH of 5. The reaction time was 24 h. The obtained materials were dried in a vacuum atmosphere at 50 °C for 24 h.

In **step 2**, reduction was performed using the thermal method. The materials were annealed at 900 °C for 5 min.

**Figure 3:**
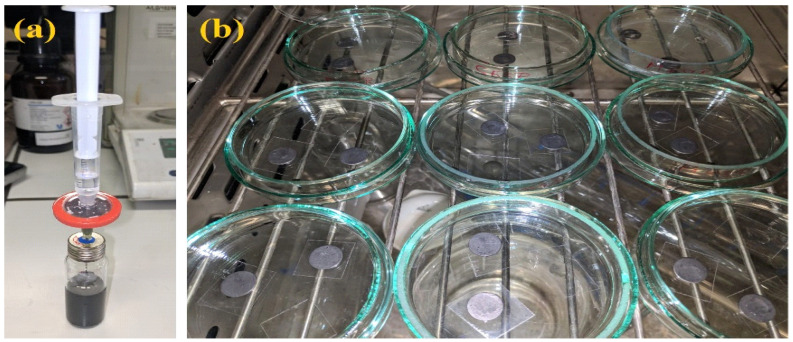
(**a**) Container with sonicated solution before filtration through a syringe filter, (**b**) rGO solution after placing on the mica surface.

In **step 3**, exfoliation was performed using sonication. The materials were sonicated in a water—N,N-dimethylformamide (DMF) 1:1 mixture with a concentration of 0.3 mg/mL of the solution. The samples were then filtered through a syringe filter (Figure 3a) and next were placed on the mica surface (Figure 3b). The reduction and exfoliation processes were the same for all samples. At the end, samples were dried initially at room temperature and then at 50 °C for 2 h.

The sensing structures were prepared using all obtained materials. The sensing materials were applied onto interdigital (IDT) transducers (substrate: SiO_2_/Si, electrodes: gold on a thin layer of chromium) using the drop-coating method. After this, all structures were annealed at 150 °C. Due to the method of application, graphene oxide was in many areas of the IDT. In the next step, all structures were placed in a measuring chamber, and the resistance of each structure was measured during the supply of various gas mixtures. The measuring station is schematically shown in [26]. Before starting the measurements, the structures were annealed at 150 °C in a dry (~5% RH) nitrogen atmosphere.

All gas sensing measurements were performed at room temperature (RT) and in dry conditions (humidity ~5%). We measured the resistance at various concentrations of nitrogen dioxide in nitrogen (0 ppm-10 ppm-0 ppm). The experiments were performed several times to obtain reliable results. Based on the obtained data, we calculated the sensitivity (S) of each structure using the following Equation (1)

(1) $$S=\frac{R_{C}-R_{M}}{R_{C}}$$

where

R_C_ is the resistance in carrier gas;

R_M_ is the resistance in a mixture of carrier gas and nitrogen dioxide.

We used Atomic Force Microscopy (NT-MDT, NTEGRA Prima platform, (NT-MDT, Eindhoven, the Netherlands, NOVA.1.1.19837 software) to evaluate the thickness of prepared reduced graphene oxides. All measurements were performed in the intermittent contact mode under air conditions with a scan rate of 0.5 Hz or 1.0 Hz depending on the sample. Since the thickness of the graphene flakes at different locations on the surface of rGOs were different, we performed 10 AFM measurements at various areas on the surface of each sample. Then, we averaged the results for each of the samples separately and calculated the number of graphene layers using the following Equation

(2) $$N_{i}=\frac{{\bar{t}}_{i}}{d+t_{m}}$$

where ${\bar{t}}_{i}$ is the average thickness of the graphene flakes calculated for a given sample, d is the distance between the graphene layers, and $t_{m}$ is the thickness of the graphene monolayer.

The Raman spectra were obtained using an N-TEGRA Spectra platform (NT-MDT, Eindhoven, the Netherlands NOVA.1.1.19837 software). A laser beam with a wavelength of 532 nm was used for the experiments, with an exposure duration of 10 s.

We assumed that the distance between the graphene layers equals 0.4 nm, and the monolayer thickness is 1.1 nm [27,28]. We determined the interplanar distance using XRD measurements. For XRD measurements, the samples were applied to glass and examined using Cu Ka1 radiation at a voltage of 45 kV and a current of 30 mA. An X’Pert PRO PW 3040/60 diffractometer (PANalytical, Quebec, QC, Canada) was used in the studies. The obtained data were then analyzed using a two-way ANOVA with interaction (to determine the influence of the graphite precursor, oxidation method, and their combination on the number of graphene layers in the prepared rGOs). This analysis was performed in Matlab R2022a.

A two-way ANOVA is generally used to determine how independent variables and their interactions affect a dependent variable. A two-way ANOVA is calculated using an F-test for statistical significance. The F-test is a group comparison test. It compares the variance for each mean of the independent variable to the overall variance of the dependent variable. For example, if the within-group variance is greater than the between-group variance, the F-test will indicate that there are no statistical differences between the tested groups. When using ANOVA, only categorical variables can be compared. Categorical variables can: be types or categories of things. More information about ANOVA can be found in [29].

In our case, we assumed the following three null hypotheses (Table 1).

## 3. Results and Discussion

**Figure 4:**
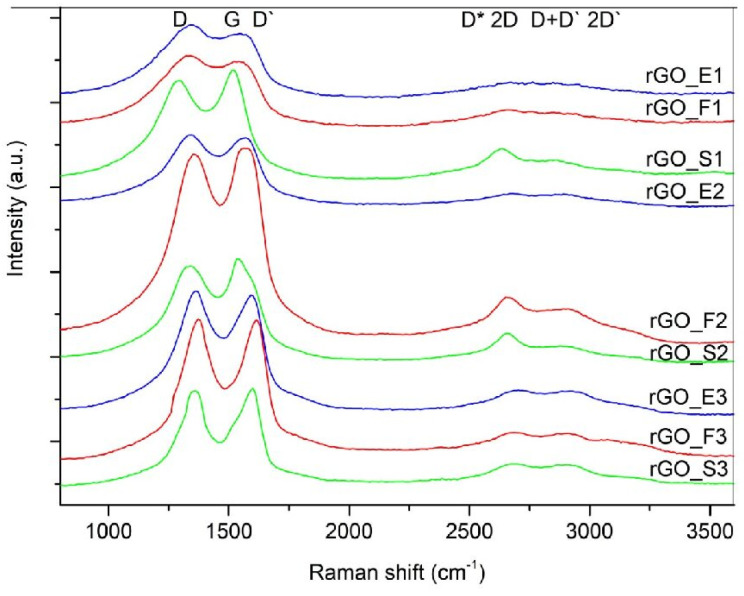
Raman spectra of reduced graphene oxides.

Reduced graphene oxides are investigated using Raman spectroscopy. The Raman spectra of the reduced graphene oxides obtained by the three methods are presented in Figure 4. These spectra show a shift of the G bands towards lower wave numbers, which is related to the lower content of oxygen groups and the recovery of the graphite structure after the applied thermal reduction step. In addition, the applied process contributes to the formation of new defects in the structure of the studied materials, as evidenced by the higher intensity of the D band. rGOs are made of graphene layers with residual oxygen groups attached to the planes and edges.

In the spectra of the rGOs, the intense G band is accompanied by a D′ band, the intensity of which is proportional to the amount of defects in the structure. The lowest intensity of the D′ band can be observed successively for samples rGO_E1, rGO_E2, rGO_S1, and rGO_S2.

**Figure 5:**
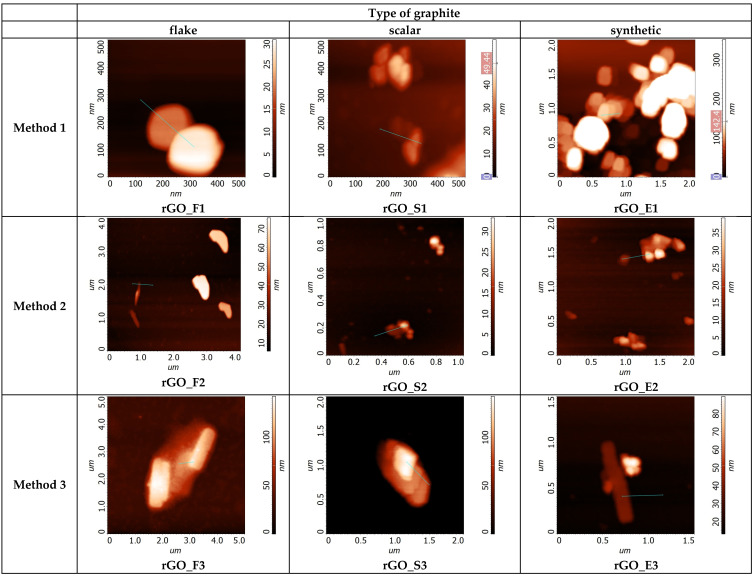
Exemplary AFM images of reduced graphene oxides obtained from various graphites and oxidized by various methods.

**Figure 6:**
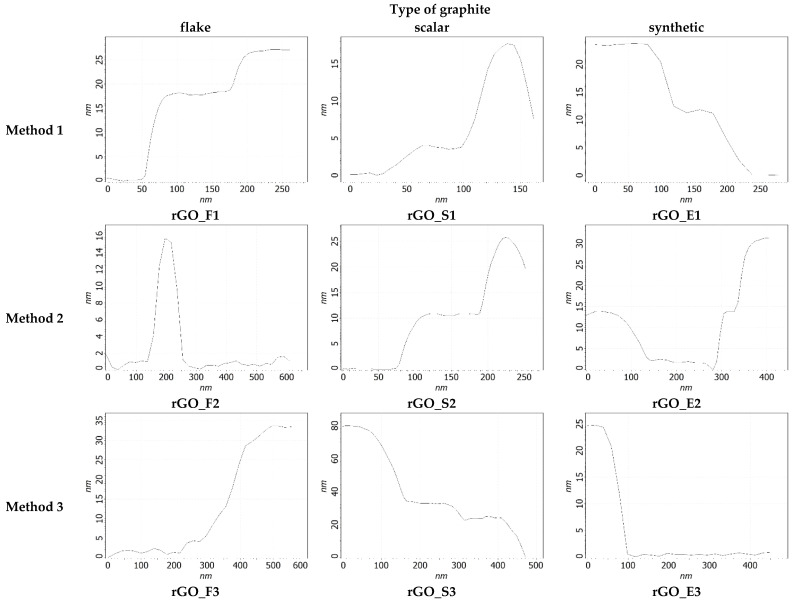
Cross-sections of reduced graphene oxides shown in Figure 5.

The next step in our investigation was the taking of measurements using atomic force microscopy. The exemplary AFM images of the investigated rGOs are shown in Figure 5, while the cross-section of these rGOs is shown in Figure 6.

**Figure 7:**
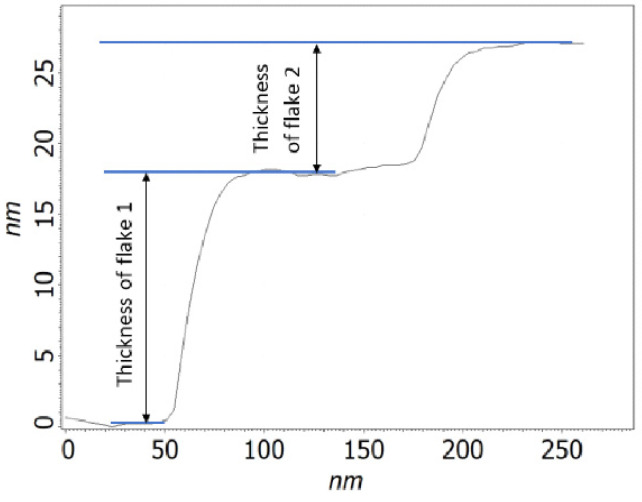
The method of the determination of the thickness of graphene flakes in the tested samples.

As can be seen, regardless of the tested material, rGO flakes have an irregular shape and different thicknesses. Therefore, the thickness of each flake was determined slightly differently. The exemplary result is shown in Figure 7.

The average values of the thickness of graphene flakes calculated for each sample and the corresponding variances and number of graphene layers (calculated from Equation (1)) are shown in Table 2.

We used these data to perform the ANOVA test. In carrying out this test, we assumed that the obtained results may be dependent on random effects. To minimize the error of random effects and interpretation errors, we applied the so-called Bonferroni correction. This correction is used to compensate for Type I error. Type I error is the likelihood of discovering a false-positive result and usually is set to 5% (α = 0.05). It consists of dividing the error by the number of comparisons, and as such, alpha is applied to a single test, e.g., when comparing two groups, significance level α = 0.05 is replaced by the value of 0.025. Then, the total error does not exceed 0.025. The results of the performed analysis are presented in Table 3.

**Figure 8:**
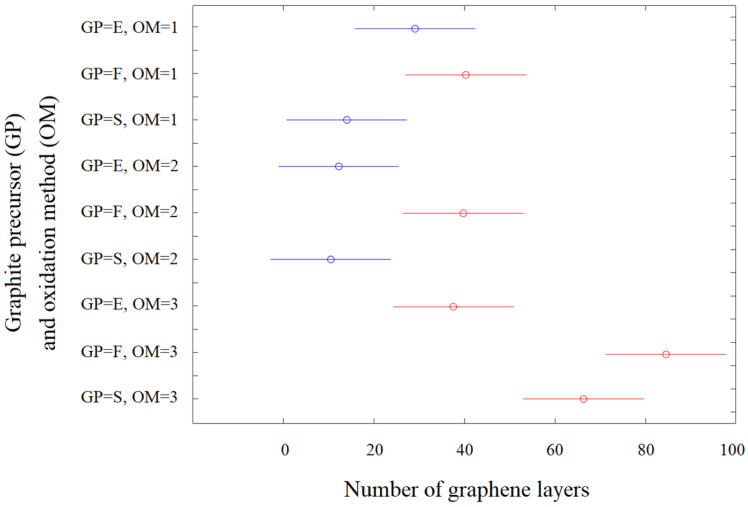
Influence of graphite precursor (GP) and oxidation method (OM) on the number of graphene layers in prepared reduced graphene oxides.

As can be seen, the *p* value of the graphite precursor is 0.0814, that of the oxidation method is 0.00250, and that of their interaction equals 0.0001. This means that the type of graphite precursor alone does not have a (statistically) significant influence on the average number of graphene layers. The much more important variable is the oxidation method, and the most important is the interaction effect between the graphite precursor and oxidation method. To determine how the analyzed variables affect the thickness of rGO flakes (how they divide/differentiate the analyzed data set in terms of the number of graphene layers), a plot of multiple comparisons was prepared (Figure 8).

**Figure 9:**
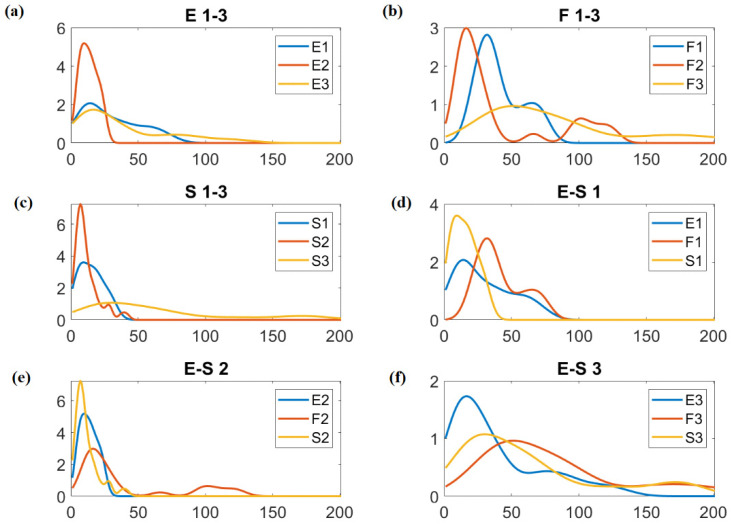
Probability distribution of the number of graphene layers in the tested samples depending on the graphite precursor and the oxidation method used. (**a**–**c**) refer to one graphite precursor and three different oxidation methods, and (**d**–**f**) refer to one oxidation method and three different graphite precursors.

Due to the possible use of the obtained materials as sensor layers (e.g., for NO_2_ detection), it was assumed that the most desirable material would be the one with the smallest number of layers [13,30,31]. Graphene oxide with a smaller number of layers has a larger specific surface area available for adsorption of molecules than multilayer graphene oxide. This criterion is met by the samples obtained from graphite S oxidized by methods 1 and 2, and graphite E oxidized by methods 1 and 2 (samples circled in blue, see Figure 8). In order to present the quality of the investigated rGOs in more detail, an analysis of the probability distribution of the obtained data (e.g., approximation of the distribution function) was performed (Figure 9). For this purpose, a distribution method was used. The distribution method is a non-parametric representation of the probability density function of a random variable. This is a good method for when we want to describe the distribution of data in more detail and avoid making assumptions about the distribution of data. The obtained data show that the third method of oxidation allows us to obtain the widest distribution of the number of layers regardless of the graphite precursor. This is undesirable from the sensor point of view (the high number of layers will limit the contact between the surface and the analyzed gas). Moreover, graphene oxide obtained from flake graphite is characterized by a wider distribution of the number of layers in comparison to graphite oxides obtained from scalar and synthetic graphite.

**Figure 10:**
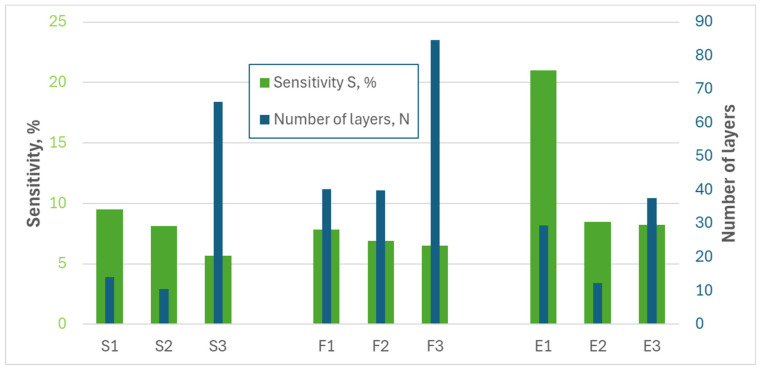
Average number of layers of analyzed materials and average sensitivity calculated for sensors with analyzed materials in an atmosphere of 10 ppm of NO_2_ in dry N_2_, in room temperature.

In order to check whether graphene oxides produced using graphite S and oxidation methods 1 and 2, as well as graphene oxides produced using graphite E and oxidation methods 1 and 2, are indeed the most suitable for detecting, e.g., NO_2_, gas measurements were carried out. For this purpose, we prepared sensing structures (chemiresistor structures) based on the tested materials and performed the experiments in various atmospheres. The obtained results are shown in Figure 10.

For all structures, the resistance decreases when NO_2_ appears in the atmosphere. The sensing mechanism of graphene oxide is based on surface adsorption of gaseous nitrogen dioxide. Reduced graphene oxide behaves like a p-type semiconductor. The change in conductivity is caused by the adsorption of NO_2_ and, as a consequence, the charge transfer and shift of the Fermi level. The resistance of the rGO decreases with increasing NO_2_ concentration [32,33].

The structures with oxide obtained from flake graphite are weakly sensitive to the oxidation method. Regardless of the graphite precursor used, the worst sensitivity was obtained for samples with materials oxidized by the third method, while the best sensitivity was obtained from the first method. The biggest sensitivity was obtained for structures with reduced/exfoliated graphene oxide marked as E1, E2, S1, and S2. The results confirmed the conclusions we had previously drawn.

**Figure 11:**
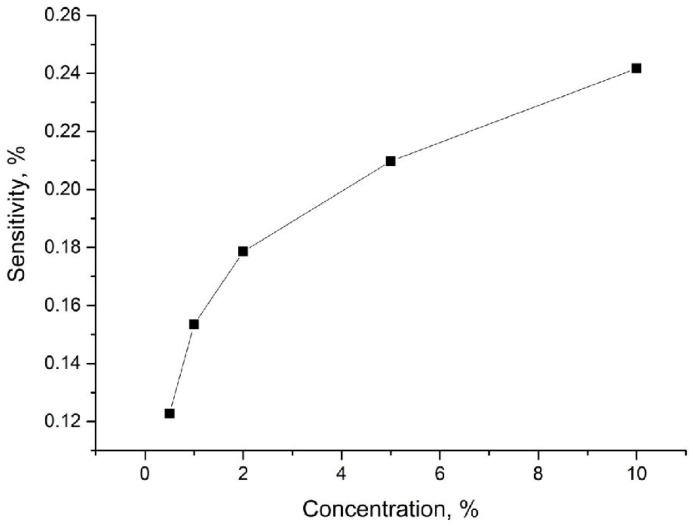
Sensitivity vs. concentration calculated for the structure with rGO_E1.

For each sensing structure, we performed additional measurements in other NO_2_ concentrations (0.5-1-2-5-10 ppm NO_2_ in N_2_). The tests were performed in room temperature and in dry (~5%) nitrogen. The exemplary results for structures with oxide are shown in Figure 11. Moreover, we calculated the response time, which is equal to ~30 min for all structures. The recovery time is longer than 1 h.

## 4. Conclusions

Reduced graphene oxide has many possible applications which are generally determined by the quality and number of rGO layers. Both of these factors depend on the fabrication process of rGO (graphite precursor, and method of oxidation, reduction, and exfoliation). Therefore, its selection is important to obtain materials with specific properties for specific purposes. However, due to the large number of possible ways of producing rGO, this is difficult. In this paper, we showed how to overcome this problem employing statistical analysis. Using ANOVA, we showed that the type of graphite precursor for a randomly selected oxidation method has no significant effect on the average number of graphene layers in rGO, whereas the selection of the appropriate oxidation method for a specific type of graphite precursor has a clear effect. It follows that the influence of the graphite precursor and the oxidation method on the number of graphene layers in rGO (and other rGO parameters) cannot be analyzed separately (as it is usually reported in the literature), but instead by taking into account both of them simultaneously. In other words, to prepare rGO with specific properties for specific applications, it is necessary to study the interaction effect between the particular variables on the rGO instead of the effect of individual variables. Unfortunately, without statistical analysis, this is difficult and time consuming. We stated that among the tested samples, the most suitable for sensor applications are the samples prepared using synthetic and scalar graphites, and the modified Hummers’ method.

## Figures and Tables

**Table 1:** Boundary conditions ANOVA hypotheses.

The Null Hypothesis (H_0_)	Alternative Hypothesis (H_a_)
Graphite precursor has no effect on the average number of graphene layers in rGO	Graphite precursor has an effect on the average number of graphene layers in rGO
Oxidation method has no effect on the average number of graphene layers in rGO	Oxidation method has an effect on the average number of graphene layers in rGO
There is no interaction effect between the type of graphite precursor and the oxidation method on the average number of graphene layers in rGO	There is an interaction effect between the type of graphite precursor and the oxidation method on the average number of graphene layers in rGO

**Table 2:** Average thickness of graphene flakes in the investigated samples and the corresponding number of graphene layers.

	rGO_E1	rGO_F1	rGO_S1	rGO_E2	rGO_F2	rGO_S2	rGO_E3	rGO_F3	rGO_S3
${\bar{t}}_{i}$ (nm)	44.2	60.4	21	18.4	59.7	15.7	56.4	126.9	99.4
σ (nm)	30.3	25.1	13.1	9.2	58.2	13.2	51.0	87.3	91.5
N	29.5	40.3	14.0	12.3	39.8	10.5	37.6	84.6	66.3

**Table 3:** Two-way ANOVA table.

Source	Sum. Sq. ^1^	d. f. ^2^	Mean. Sq. ^3^	F ^4^	Prob > F(*p*) ^5^
Graphite precursor	161,631.1	2	80,815.6	5.01	0.0814
Oxidation method	341,428.9	2	170,714.4	10.58	0.0250
Interaction	64,513.5	4	16,128.4	6.05	0.0001

^1^ Sum. Sq. sum of squares, ^2^ d. f. degree of freedom for each variable, ^3^ Mean. Sq. mean sum of squares, ^4^ F test statistic from F-test, ^5^ Prob > F(*p*) *p* value of the statistic.

## Data Availability

Data are contained within the article.
